# Ribosomal DNA transcription in the dorsal raphe nucleus is increased in residual but not in paranoid schizophrenia

**DOI:** 10.1007/s00406-014-0518-4

**Published:** 2014-08-05

**Authors:** Marta Krzyżanowska, Johann Steiner, Ralf Brisch, Christian Mawrin, Stefan Busse, Katharina Braun, Zbigniew Jankowski, Hans-Gert Bernstein, Bernhard Bogerts, Tomasz Gos

**Affiliations:** 1 Department of Forensic Medicine, Medical University of Gdańsk, Ul. Dębowa 23, 80-204 Gdańsk, Poland; 2 Department of Psychiatry, Otto-von-Guericke-University, Magdeburg, Germany; 3 Institute of Neuropathology, Otto-von-Guericke-University, Magdeburg, Germany; 4 Department of Zoology/Developmental Neurobiology, Institute of Biology, Otto-von-Guericke-University, Magdeburg, Germany

**Keywords:** Postmortem, Schizophrenia, Paranoid, Residual, Dorsal raphe nucleus, AgNOR staining

## Abstract

The central serotonergic system is implicated in the pathogenesis of schizophrenia, where the imbalance between dopamine, serotonin and glutamate plays a key pathophysiological role. The dorsal raphe nucleus (DRN) is the main source of serotonergic innervation of forebrain limbic structures disturbed in schizophrenia patients. The study was carried out on paraffin-embedded brains from 17 (8 paranoid and 9 residual) schizophrenia patients and 28 matched controls without mental disorders. The transcriptional activity of ribosomal DNA (rDNA) in DRN neurons was evaluated by the AgNOR silver-staining method. An increased rDNA transcriptional activity was found in schizophrenia patients in the cumulative analysis of all DRN subnuclei (*t* test, *P* = 0.02). Further subgroup analysis revealed that it was an effect specific for residual schizophrenia versus paranoid schizophrenia or control groups (ANOVA, *P* = 0.002). This effect was confounded neither by suicide nor by antipsychotic medication. Our findings suggest that increased activity of rDNA in DRN neurons is a distinct phenomenon in schizophrenia, particularly in residual patients. An activation of the rDNA transcription in DRN neurons may represent a compensatory mechanism to overcome the previously described prefrontal serotonergic hypofunction in this diagnostic subgroup.

## Introduction

The central serotonergic system is implicated in the pathogenesis of numerous mental disorders, including schizophrenia, where the imbalance between dopamine, serotonin and glutamate plays a key pathophysiological role [for reviews see: 1, 24, 49]. As revealed by neuropathological research on depression and suicide, abnormalities in the serotonergic system may be structurally restricted to a specific brain region, the dorsal raphe nucleus (DRN), which affects brain circuits [for a review see: 5]. DRN neurons provide the major serotonergic innervation to the prefrontal cortex (PFC) [42, 72, 78]. Limbic regions of the PFC [i.e. the anterior cingulate cortex (AC) and the orbitofrontal cortex (OFC)] may in turn reciprocally regulate DRN function through glutamatergic pyramidal efferents [31, 44, 45, 52]. This output is controlled by serotonergic receptors on both pyramidal cells and cortical interneurons [64, 76]. Among them, the 5HT2A receptors on pyramidal neurons play an outstanding role as the primary sites of action of hallucinogens and atypical neuroleptics [for reviews see: 1, 24, 49].

However, compared with the number of postmortem studies on the DRN in affective disorders and suicide [2, 4, 6, 7, 9, 12–17, 33, 46, 47, 69, 71], reports on the DRN in schizophrenia are scanty and they have not revealed specific abnormalities for this diagnostic entity [22, 47, for a review of older studies see: 39]. Moreover, the neuropathological studies of DRN have not addressed the differentiation between paranoid and residual schizophrenia, which are substantial diagnostic subgroups with distinct clinical presentations (the former being characterised by predominantly psychotic and the latter by predominantly negative symptoms) and distinct abnormalities in the serotonergic system [for reviews see: 1, 49].

Nucleolar organising regions (NORs) are genetic loci on chromosomes that are composed of ribosomal DNA (rDNA) and proteins, some of which are argyrophilic. In human interphase cells, silver-stained NORs (AgNORs) clustered together in the nucleolus represent the site of transcriptionally active NORs and ribosomal RNA synthesis, which constitutes approximately one half of the entire transcriptional activity in the cell. In the AgNOR staining evaluated by light microscopy, AgNORs are indistinguishable from each other and form the AgNOR area, located in the nucleolar area but smaller than this area (compared, for instance, with haematoxylin–eosin and Nissl stainings, [50]). As a surrogate marker of protein biosynthesis and an important sensor of cellular stress of different nature, the transcriptional activity of rDNA can be assessed by measuring AgNOR parameters. These are AgNOR area (representing the nucleolus), AgNOR number (i.e. the number of AgNOR areas within one nucleus) and AgNOR ratio defined as the quotient of total AgNOR area in the nucleus and nuclear area [12, 25, 29, 32–37, 40, 54–56, 62; for reviews see: 59, 65].

A key role of rDNA transcriptional activity in neuronal plasticity has been proven in neuronal culture [30], and molecular studies have revealed that this activity is disturbed in cortical areas in mental disorders [26, 27, 48, 53]. Our previous AgNOR studies of the DRN [33] and other brain structures [32, 34–37] in depression have suggested disturbed (predominantly decreased) rDNA transcription in neurons specifically in suicidal patients, which is consistent with molecular results [48]. In the present study, we hypothesised the existence of differences in the transcriptional activity of rDNA in DRN neurons specific for schizophrenia patients from both paranoid and residual diagnostic subgroups and tested this hypothesis by the application of the AgNOR staining method in our postmortem material.

## Materials and methods

### Human brain tissue

Brains of controls and patients with established diagnoses of paranoid and residual schizophrenia according to the DSM-IV criteria, and no history of substance abuse, were obtained in accordance with existing EU regulations from the Magdeburg Brain Bank. Demographic, clinical and histological data are summarised in Table 1. The predominance of suicide in the paranoid subgroup of our cohort is consistent with clinical reports, which emphasise the role of positive symptoms as a suicide risk factor in schizophrenia [41]. Qualitative neuropathological changes suggestive of vascular, traumatic, inflammatory, neoplastic or neurodegenerative processes were excluded by an experienced neuropathologist (C.M.). All the patients who had received antipsychotic medication during the 90 days prior to death were treated with typical antipsychotics. The mean doses of antipsychotic medication in the last 90 days of life were established from the clinical records, taking into consideration the reported chlorpromazine equivalents [3, 61].

**Table 1:** Demographic data of patients with schizophrenia (*n* = 17) and healthy control subjects (*n* = 28)

Case ID	Diagnosis (DSM-IV)	Sex	Age (year)	Illness duration (year)	Autolysis time (h)	CPZ (mg)	Cause of death
1	Sz, paranoid	F	49	2	72	300	Fall from the height
2	Sz, paranoid	M	47	16	24	785	Strangulation
3	Sz, paranoid	F	52	28	24	n.a.	Drowning
4	Sz, paranoid	F	55	6	48	n.a.	Intoxication
5	Sz, paranoid	M	34	5	5	n.a.	Hanging
6	Sz, paranoid	M	45	20	72	740	Tracheobronchitis
7	Sz, paranoid	M	38	16	24	505	Self-strangulation
8	Sz, paranoid	M	27	5	24	150	Hanging
9	Sz, residual	M	46	18	48	846	Pulmonary embolism
10	Sz, residual	M	51	28	48	758	Ileus
11	Sz, residual	M	57	23	72	n.a.	Cardiac insufficiency
12	Sz, residual	F	64	0	12	n.a.	Pulmonary embolism
13	Sz, residual	M	48	32	48	n.a.	Acute respiratory failure
14	Sz, residual	M	58	25	24	n.a.	Cardio-respiratory insufficiency
15	Sz, residual	M	54	34	48	n.a.	Cardio-respiratory insufficiency
16	Sz, residual	F	54	18	24	600	Pulmonary embolism
17	Sz, residual	M	76	26	12	0	Self-strangulation
Sz, all cases (ratio/mean ± SD)		12M/5F	50 ± 11	18 ± 11	37 ± 22	520 ± 304	
Sz, paranoid (ratio/mean ± SD)		5M/3F	43 ± 10	12 ± 9	37 ± 25	496 ± 274	
Sz, residual (ratio/mean ± SD)		7M/2F	56 ± 9	23 ± 10	37 ± 20	551 ± 381	
18	Control	M	56	–	48	–	Retroperitoneal haemorrhage
19	Control	M	47	–	24	–	Myocardial infarction
20	Control	F	52	–	24	–	Cardio-respiratory failure
21	Control	F	48	–	48	–	Status asthmaticus
22	Control	M	47	–	24	–	Acute respiratory failure
23	Control	F	64	–	24	–	Peritonitis
24	Control	F	33	–	72	–	Aortic embolism
25	Control	M	40	–	96	–	Myocardial infraction
26	Control	M	64	–	35	–	Ruptured aortic aneurysm
27	Control	F	48	–	26	–	Pulmonary embolism
28	Control	F	65	–	24	–	Heart failure
29	Control	F	30	–	48	–	Pulmonary embolism
30	Control	M	56	–	24	–	Pulmonary embolism
31	Control	F	64	–	26	–	Myocardial infarction
32	Control	M	63	–	48	–	Heart failure
33	Control	F	61	–	8	–	Heart failure
34	Control	F	61	–	24	–	Heart failure
35	Control	F	38	–	24	–	Heart failure
36	Control	M	39	–	4	–	Peritonitis
37	Control	F	61	–	24	–	Heart failure
38	Control	F	67	–	24	–	Sudden cardiac death
39	Control	M	54	–	24	–	Pulmonary embolism
40	Control	M	46	–	24	–	Lymphoma
41	Control	F	63	–	24	–	Myocardial infarction
42	Control	F	39	–	48	–	Heat stroke
43	Control	F	66	–	24	–	Right heart failure
44	Control	F	39	–	48	–	Myocardial infarction
45	Control	F	50	–	72	–	Ruptured aortic aneurysm
Controls (ratio/mean ± SD)		10M/18F	52 ± 11	–	34 ± 20	–	

Test		Chi-square	ANOVA	–	ANOVA	–	
*Statistic*							
Sz, paranoid/residual versus controls							
Characteristic value		*χ* ^2^ = 5.544	*F* = 3.428	–	*F* = 0.084	–	
*P* value		0.063	**0.042**	–	0.920	–	

Test		Chi-square	*t* test	*t* test	*t* test	*t* test	
*Statistic*							
Sz, paranoid versus Sz, residual							
Characteristic value		*χ* ^2^ = 0.476	*T* = −2.882	*T* = −2.216	*T* = −0.065	*T* = −0.253	
*P* value		0.490	**0.011**	**0.043**	0.949	0.808	

*Sz* schizophrenia, *f* female, *m* male, *SD* standard deviation, *n.a.* no available data, *CPZ* chlorpromazine equivalents of the mean dose of antipsychotic medication taken by the patients during the last 90 lifetime days; significant *P* values are in bold

**Fig. 1:**
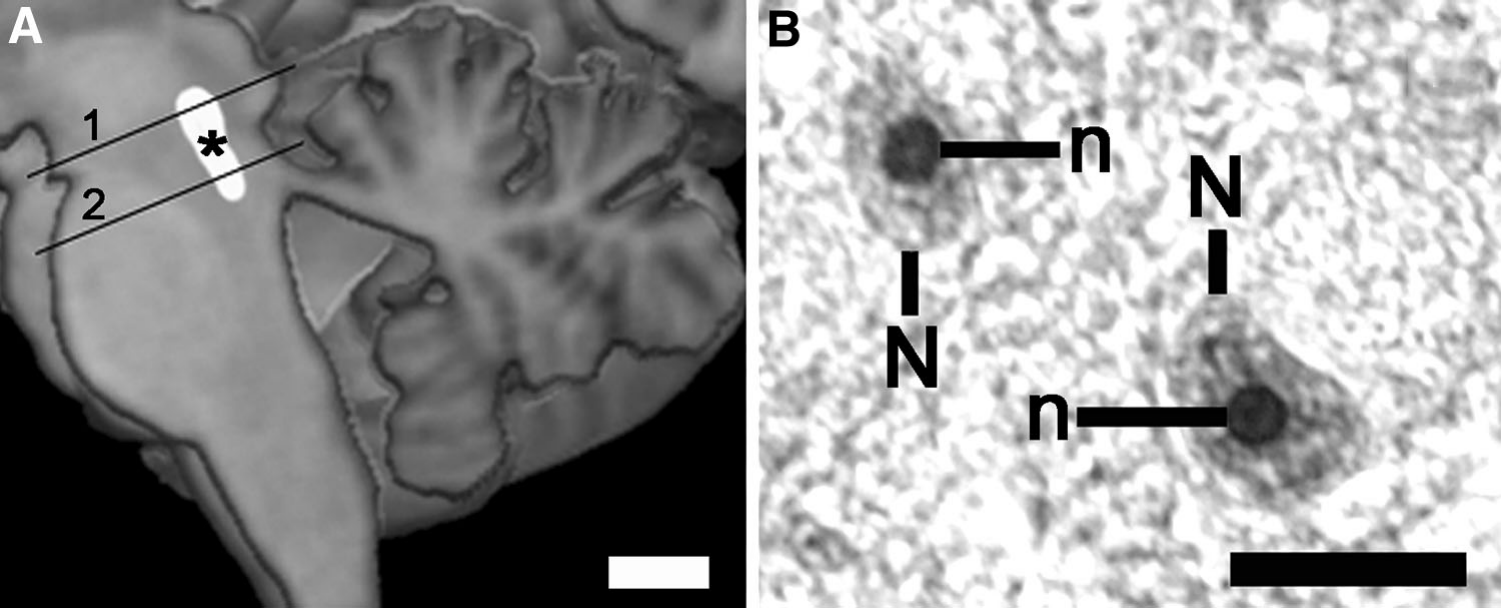
**a** Schematic picture of dorsal raphe nucleus localisation (*) with both levels (1, 2) where the investigations are carried out (*scale bar* 10 mm). **b** After AgNOR staining, the borders of AgNOR areas (representing nucleoli) (*n*) are clearly visible within nuclei (*N*) of DRN neurons (residual schizophrenia case, ventral subnucleus, *scale bar* 20 µm). The differences in AgNOR parameters are beyond qualitative evaluation and they could only be captured by means of quantitative measurements

The tissue preparation was performed as previously described [33]. Briefly, brains were fixed in toto in 8 % phosphate-buffered formaldehyde for at least 2 months (pH = 7.0; temperature 15–20 °C). The brainstem was isolated by a cut made perpendicularly to its longitudinal axis at the point of emergence of the oculomotor nerve. A second transverse cut was made at the caudal level of the medulla. After being embedded in paraffin, serial 20-µm-thick transverse sections were cut along the entire rostrocaudal axis of the brainstem and mounted. Every 50th section was Nissl (cresyl violet) and myelin (Heidenhain-Wölcke) stained. The first rostral section of the DRN stained for AgNOR was adjacent to the one randomly selected from the first three Nissl-stained sections of the rostral DRN at the level of the trochlear nucleus. Accordingly, the first caudal section of the DRN was selected at the level of the rostral locus coeruleus. Thus, the selection of sections for AgNOR staining was in accordance with the principle of systematic sampling. Subsequently, one additional section from the rostral and caudal DRN was stained for AgNOR. The distance between two sections in the rostral as well as in the caudal DRN was 1 mm. Consequently, two sections at the level of the trochlear nucleus, containing the ventral, ventrolateral, dorsal and interfascicular subnuclei, and two sections at the level of the rostral locus coeruleus, containing the caudal subnucleus of the DRN (Fig. 1a), were used for the evaluation of AgNOR parameters in each of the investigated cases.

### AgNOR staining

Silver staining was carried out as previously described [33]. Briefly, 20-µm paraffin sections were dewaxed and rehydrated through graded alcohols. The silver staining was freshly prepared by dissolving 2 g/dl gelatin in 1 ml/dl aqueous silver nitrate solution at a 1:2 ratio. Sections were incubated with this solution in a dark moist chamber at room temperature for 45 min followed by washing with deionised water. [The section thickness after the histological procedures was 16.7 ± 1.7 µm (mean ± SD)] Following this protocol, the AgNOR area—containing AgNORs (that are clustered, undistinguishable from each other) and representing the nucleolus—appears as an intranuclear, clearly delineated black or dark brown small spot and the nuclear border is clearly visible in the majority of large DRN neurons, which are most probably serotonergic output neurons [8]. Glial cells were distinguished from neurons according to established cytomorphological criteria [57].

### Quantification

The anatomical borders of the DRN subnuclei were determined according to the description by Baker et al. [8]. In each of the five DRN subnuclei (i.e. the dorsal, ventrolateral, ventral, interfascicular and caudal), AgNOR parameters were determined in 40 neurons (20 in each of the two sections) with clearly visible borders of the nucleus and AgNOR areas. Thus, the AgNOR parameters of 200 DRN neurons were investigated in each of the cases. The number of the investigated neurons was established arbitrarily by us in accordance with the guidelines on quantitative evaluation [38] and the cited diagnostic and research studies employing the AgNOR method. This method does not require morphometric evaluation of the number (of cells and/or nuclei) or the volume (of nuclei and intranuclear structures) [25, 54–56, 62].

The AgNOR areas (composed of clustered AgNORs and representing the nucleoli), their number and the nuclear area within a single-sampled neuron were determined using a light microscope attached to a computer image analysis system (cellSens^®^ Standard, Olympus, Japan). In this system, each of the neurons sampled by 400× magnification was visualised digitally, focused, and the sharpest and longest profiles of the nucleus and AgNOR area were traced by the mouse cursor on the screen. As a result, numerical values of AgNOR and nuclear areas and the numbers of AgNOR areas were calculated automatically. Subsequently, the AgNOR ratio (relative AgNOR area) was derived by dividing the total AgNOR area by the nuclear area, taking into account all the AgNOR areas present per neuronal nucleus, which were shown by careful focusing within the nuclear area. This procedure was performed separately for each of the sampled neurons. The sampled measures were averaged to derive single values for each DRN subnucleus, the rostral and caudal subdivisions of the DRN and the entire DRN as a single anatomical structure in the investigated individual.

Measurements of the nuclear area and the AgNOR area, as well as the counting of AgNOR areas, were performed by one of the authors (M.K.) blinded to the diagnosis. To establish the interrater (M.K., T.G.) and the test–retest reliabilities, repeated measurements for 5 brains were carried out. Intraclass correlation analyses yielded highly similar values of the investigated parameters with the *r* value ranging from 0.92 to 0.97. The interrater reliability was in the *r* range of 0.93–0.98.

### Data analysis

Statistical analyses were performed with the data analysis software system STATISTICA version 10 (StatSoft^®^, Inc. 2011, www.statsoft.com). The AgNOR data were normally distributed, as indicated by the Kolmogorov–Smirnov test. Therefore, the *t* test and the analysis of variance (univariate ANOVA) followed by post hoc comparisons with the *t* test were employed to analyse the AgNOR data. The chi-square test, the *t* test and the ANOVA were used to detect the possible differences between the study groups with respect to age, sex, duration of the disease, season of the year (month of death in spring/summer vs. autumn/winter), postmortem interval, suicide and medication dosage. Due to their greater robustness, nonparametric tests (the Mann–Whitney *U* test and the Kruskal–Wallis *H* test) were additionally used to confirm the diagnosis-dependent results. All statistical tests were two tailed. The Pearson correlation coefficient and the Spearman rank correlation coefficient were calculated to determine the influence of the above demographic, clinical and methodological variables which might confound the results for the dependent variables. Generally, significance was defined as *P* < 0.05 (with the Bonferroni correction for multiple comparisons).

## Results

### Qualitative analysis of the AgNOR staining

After AgNOR staining of the DRN neurons, borders of the AgNOR area (containing AgNORs that were clustered together and indistinguishable from each other) and the nucleus were clearly visible (Fig. 1) in line with the staining patterns in DRN neurons presented previously [33]. Most of the neurons contained one AgNOR area (representing the nucleolus in this staining method). Two or more AgNOR areas were observed very rarely, which explains why the AgNOR number was near 1.

### Quantitative analysis of the AgNOR staining

The differences in AgNOR parameters were beyond qualitative evaluation, and they could only be captured by means of quantitative measurements.

The statistical analysis revealed significant differences by means of the cumulative analysis of all DRN subnuclei. An increased AgNOR area was observed in schizophrenia patients compared to controls (21.19 ± 3.69 vs. 19.01 ± 2.39 µm^2^, respectively; *t* test *P* = 0.02).

As summarised in Table 2, a further subgroup analysis revealed an increase of AgNOR area in the residual schizophrenia versus paranoid schizophrenia or control groups (ANOVA, *P* = 0.002).

**Table 2:** AgNOR parameters in the global analysis of all DRN subregions in paranoid versus residual schizophrenia patients and controls

Parameter	*C*, *n* = 28	*P*, *n* = 8	*R*, *n* = 9	*P* values	*P* values	*P* values
	Mean ± SD	Mean ± SD	Mean ± SD	*P* versus *R*	*P* versus *C*	*R* versus *C*
Nuclear area in µm^2^	108.8 ± 28.5	107.4 ± 22.7	112.9 ± 27.4	n.s.	n.s.	n.s.
AgNOR area in µm^2^ per nucleus	19.01 ± 2.39	19.27 ± 2.12	22.9 ± 4.04	**0.028**	n.s.	**0.002**
Number of AgNORs per nucleus	1.06 ± 0.06	1.03 ± 0.03	1.07 ± 0.07	n.s.	n.s.	n.s.
Relative AgNOR area	0.191 ± 0.029	0.189 ± 0.020	0.230 ± 0.034	**0.030**	n.s.	**0.007**

*C* controls, *P* paranoid schizophrenia, *R* residual schizophrenia, *n.s.* non-significant, *n* number of cases, *SD* standard deviation, *P values* *t* tests *P* values (with Bonferroni correction for multiple comparisons; significant values are in bold)

### Confounders

No significant correlations were found between the mean daily doses of antipsychotic medication given in the 90 days before death and the AgNOR parameters in the cumulative analysis of the DRN, either in the entire schizophrenic cohort or in the diagnostic subgroups. Other variables which could influence the AgNOR parameters in the DRN neurons, such as suicide, postmortem interval, sex, age at death, season of the year at death or duration of disease, were also not correlated with the values of the AgNOR parameters in the compared groups.

The age at death was significantly lower in paranoid versus both residual subgroup and controls (ANOVA, *P* = 0.042), the duration of disease was significantly longer in residual compared with paranoid patients (*t* test, *P* = 0.043), and suicides unequivocally prevailed in paranoid versus residual subgroup (7 of 8 compared with 1 of 9, respectively; chi-square test *P* = 0.002). However, these factors did not correlate with any of the AgNOR parameters. Therefore, they were not included as covariates in the analysis of covariance.

## Discussion

Our study has revealed a significantly increased AgNOR area in DRN neurons suggestive of their increased rDNA transcriptional activity in schizophrenia patients versus controls, particularly in the subgroup of patients with residual schizophrenia. The observed effect was not confounded by other variables, including antipsychotic medication and suicide. The latter point differs from our previously published findings in depression, which had suggested decreased rDNA transcription in DRN neurons specific for suicide in relation to self-aggression level [33]. Moreover, in contrast to our present results, other postmortem [22, 47, for a review of older studies see: 39] and neuroimaging research [for reviews see: 1, 28] did not reveal abnormalities in the DRN of schizophrenia patients. The discrepancy may result from various factors; however, the different methodology, inconsistencies in psychiatric diagnosis and treatment, and small sample sizes seem to be the most relevant.

The statistical significance was shown in the cumulative analysis of all DRN subnuclei. The observed effect could be related to the characteristics of DRN efferents which overlap in target structures in spite of the accentuated specialisation [42, 72, 75, 78].

To the best of our knowledge, the present AgNOR study of the DRN is the first one to have been performed on human material in schizophrenia. However, the interpretation of our results is not unequivocal due to the very sophisticated nature of DRN activity regulation [21, 64, 66, 73]. Tracing studies consistently reveal that the limbic PFC and the habenula, an evolutionarily conserved link between the forebrain and the midbrain, are the main sources of DRN afferents [10, 31, 44, 45, 52]. However, the net effect of their excitatory glutamatergic inputs consists in the inhibition of DRN neurons, as they activate predominantly local GABAergic neurons [44, 64, 74]. Therefore, the disturbed inputs from both regions and/or their disturbed local transformation in the DRN play most probably an important role in the observed difference between residual and paranoid patients.

This effect may be related to the serotonergic hypofunction in PFC areas in cases with a predominantly negative symptomatology. This hypofunction has been mainly suggested by many consistent postmortem reports on the increased density of the postsynaptic 5HT1A receptors unrelated to the cause of death [for reviews see: 1, 28]. Disturbances in the 5HT2A receptors have also been consistently reported. However, the studies on these receptors have provided partially contradictory findings, possibly a result of cohort composition, medication status and assay methodology [23, 51, 60, for reviews see: 1, 28, 49]. A decreased density of these receptors in the PFC has been suggested to be specific for non-suicidal residual schizophrenia patients [1], who predominated (8 out of 9) in our residual subgroup.

The major cortical cell type that expresses the 5HT2A receptors is the pyramidal neuron [43, 77]. In experimental models, the hypofunction of these receptors on pyramidal neurons in the limbic PFC provides their decreased output with subsequently decreased activity of local GABAergic inhibitory interneurons in the DRN and thus the disinhibition of DRN neurons [64]. Taking into consideration the results of neuronal cultures [11, 30, 79], one consequence of attenuated GABAergic inhibition of these neurons may be their increased rDNA transcriptional activity. Therefore, the hypothetically decreased 5HT2A receptors on pyramidal neurons in the limbic PFC in residual schizophrenia with subsequent hypoactivity of these neurons might lead to the increased rDNA transcription in DRN neurons suggested by our study. The concept of chronic pyramidal hypofunction in limbic PFC areas in schizophrenia has been supported more directly by the neuropathological research on human brain, which revealed decreased neuronal body size [18, 20] and dendrites reduction in AC pyramidal neurons [19]. Correspondingly, structural magnetic resonance imaging (MRI) revealed OFC white matter deficits in patients with predominantly negative symptoms [63] in accordance with the results of neuropathological research suggestive of oligodendrocytes hypofunction in residual schizophrenia [67, 68]. These findings support a hypothesis that decreased pyramidal output from limbic PFC areas may be a phenomenon accentuated in residual patients. Also, recent structural MRI reports suggest a relationship between negative symptomatology in schizophrenia and deficits in limbic prefrontal regions [58, 70], which directly regulate DRN function [31, 44, 45, 52, 73, 76]. Therefore, the increased rDNA transcription in DRN neurons suggested by our results in residual schizophrenia may be a consequence of prefrontal serotonergic hypofunction (via decreased pyramidal output), which was probably more accentuated in patients with predominantly negative symptoms in our cohort.

### Limitations

The present study has certain limitations that need to be considered: (1) As is usually the case in postmortem analyses, the reported data are cross-sectional, while longitudinal data would be ideal to clarify whether the diagnostic subgroups of residual and paranoid schizophrenia represent two different endophenotypes with a distinct neurobiology or two different disease stages. At this point, it is impossible for us to determine whether the observed changes mirror the trait versus state marker framework. Moreover, because the duration of the disease had no significant influence on our results, we were unable to identify a disease-stage related pattern. (2) We have analysed a relatively small number of cases and our findings would need to be confirmed in a larger sample. (3) An influence of antipsychotic medication on the outcome of our study cannot be excluded because no reliable data on psychotropic medication for a period beyond the 3 months prior to death were available. (4) We have previously noted that AgNOR abnormalities in the DRN may also reflect a distinct neurobiology of suicide. According to our statistical analysis, the factor “suicide” is unlikely to confound our results despite of the predominance of suicidal cases in the paranoid schizophrenia subgroup. Nevertheless, an extended analysis of additional suicide cases would be necessary to obtain concluding evidence regarding this issue.

## Conclusion

In conclusion, our results suggest increased rDNA activity in DRN neurons of patients with residual schizophrenia as a presumable consequence of prefrontal serotonergic hypofunction in this diagnostic subgroup. If this finding is consistently present in patients with more prominent negative symptoms compared to those with prominent positive symptoms, the observed phenomenon might be useful to identify distinct disease endophenotypes within the spectrum of psychotic disorders, which are currently classified as schizophrenia.
